# P111 Recurrent Pleural Effusion in an 11-Year-Old: A Diagnostic and Therapeutic Challenge

**DOI:** 10.1093/rap/rkag113.141

**Published:** 2026-11-03

**Authors:** Suchika Garg, Anushka Prabhudesai, Archana Khan, Raju Khubchandani

**Affiliations:** North Middlesex University Hospital, London, United Kingdom; Department of Pediatric Rheumatology, NH SRCC Children’s Hospital, Mumbai, India; Department of Pediatric Rheumatology, NH SRCC Children’s Hospital, Mumbai, India; Department of Pediatric Rheumatology, NH SRCC Children’s Hospital, Mumbai, India; Department of Pediatric Rheumatology, NH SRCC Children’s Hospital, Mumbai, India

## Abstract

**Introduction:**

Recurrent pleural effusion in children is uncommon and often reflects an underlying multisystem disorder. Thoracic involvement with interstitial or mediastinal disease can cause significant morbidity, and diagnosis is frequently delayed due to nonspecific symptoms and overlapping differentials. Targeted therapies have shown benefit in selected complex lymphatic disorders, particularly with mTOR inhibition.

**Case description:**

An 11-year-old girl presented with respiratory distress and hypoxia (SpO₂ 88% room air). She had recurrent pleural and pericardial effusions dating back to 2015, including an episode of cardiac tamponade requiring urgent drainage. Multiple admissions between 2019–2021 documented persistent pleural collections. Pleural fluid was repeatedly exudative with raised triglycerides; cytology showed lymphocyte-predominant effusion but no malignant cells. TB work-up (GeneXpert, AFB) was consistently negative. Bone marrow biopsy was normocellular with mild reticulin increase; flow cytometry showed polyclonal T-cell predominance.

Serial imaging revealed a progressive multisystem infiltrative process: hepatosplenomegaly with multiple splenic lesions, retroperitoneal and para-aortic lymphadenopathy, vertebral body abnormalities without cortical destruction, diffuse interlobular septal thickening, peribronchovascular infiltration, and sheet-like mediastinal lesions extending into the neck. Early spirometry showed small airway obstruction and moderate restriction. The constellation of thoracic, abdominal, and skeletal findings raised suspicion for a complex lymphatic anomaly, including generalised lymphatic anomaly (GLA) as a key differential.

Given recurrent effusions, progressive thoracic disease, and nondiagnostic investigations, targeted therapy with sirolimus was initiated (0.8 mg/m²; trough 10–15 ng/mL). After one year, she had complete resolution of dyspnoea, normal oxygen saturation, and no hospitalisations. Spirometry improved markedly (FEV1 46%→65%; FVC 46%→61%). She completed a 6-minute walk test (486 m, SpO₂ 100%). Follow-up MRI showed regression of vertebral and splenic lesions, marginal reduction in pericardial thickening, and stable thoracic infiltration. Hyperlipidaemia developed, requiring statin therapy.

**Discussion:**

This case illustrates the complexity of evaluating recurrent effusions in children, where infection, malignancy, and autoimmune disease were excluded despite extensive testing. The chronicity of lymphocyte-predominant, triglyceride-rich effusions, combined with diffuse thoracic infiltration and multifocal skeletal and splenic lesions, strongly suggested an underlying complex lymphatic disorder, with GLA being a leading consideration. Longitudinal imaging was crucial in recognising the multisystem pattern typical of lymphatic anomalies.

The substantial functional and radiological improvement following mTOR inhibition supports emerging evidence for sirolimus in GLA and related lymphatic anomalies, where mixed responses across organ systems are common. This case emphasises the importance of considering lymphatic disorders early, especially when conventional investigations are unrevealing, and highlights the need for structured monitoring due to metabolic adverse effects.

**Key learning points:**

• Consider complex lymphatic disorders, including GLA, in children with recurrent exudative pleural or pericardial effusions and raised triglycerides.

• Longitudinal imaging can reveal evolving multisystem involvement notapparentinitially.

• Nondiagnostic cytology and bone marrow biopsy do not exclude rare infiltrative lymphatic disorders.

• Vertebral lesions without cortical destruction may suggest lymphatic infiltration rather than malignancy.

• MRI is more sensitive than CT for mediastinal, splenic, and skeletal involvement.

• Functional tests (spirometry, 6MWT) provide objective markers of response.

• Targeted mTOR inhibition can significantly improve thoracic disease burden in suspected lymphatic anomalies.

• Monitoring trough levels, lipids, liver function, and blood counts is essential for children started on mTOR inhibitors.

